# 

**Published:** 1967-04

**Authors:** 


					April 1967
Medico*
national library oe medicin
RE00' APR 2 4 1967 '
SHELVED IN RR: YES.
J ? -L NO.:
rjY^Y NO. -
U ? =
L?jjE.;:;ER:
INCORPORATING THE MEDICAL JOURNAL OF THE SOUTH
Vol 82 (ii) No 304

				

